# Immunomodulatory Properties of Carvone Inhalation and Its Effects on Contextual Fear Memory in Mice

**DOI:** 10.3389/fimmu.2018.00068

**Published:** 2018-01-25

**Authors:** Aritz Lasarte-Cia, Teresa Lozano, Marta Pérez-González, Marta Gorraiz, Kristina Iribarren, Sandra Hervás-Stubbs, Pablo Sarobe, Obdulia Rabal, Mar Cuadrado-Tejedor, Ana García-Osta, Noelia Casares, Juan José Lasarte

**Affiliations:** ^1^Immunology and Immunotherapy Program, Center for Applied Medical Research (CIMA), University of Navarra, Instituto de Investigación Sanitaria de Navarra (IdiSNA), Pamplona, Spain; ^2^Neuroscience Program, Center for Applied Medical Research (CIMA), University of Navarra, Instituto de Investigación Sanitaria de Navarra (IdiSNA), Pamplona, Spain; ^3^Small Molecule Discovery Platform, Center for Applied Medical Research (CIMA), University of Navarra, Instituto de Investigación Sanitaria de Navarra (IdiSNA), Pamplona, Spain; ^4^Anatomy Department, School of Medicine, University of Navarra, Pamplona, Spain

**Keywords:** immunomodulation, odours, olfactory system, central nervous system, memory

## Abstract

A complex network of interactions exists between the immune, the olfactory, and the central nervous system (CNS). Inhalation of different fragrances can affect immunological reactions in response to an antigen but also may have effects on the CNS and cognitive activity. We performed an exploratory study of the immunomodulatory ability of a series of compounds representing each of the 10 odor categories or clusters described previously. We evaluated the impact of each particular odor on the immune response after immunization with the model antigen ovalbumin in combination with the TLR3 agonist poly I:C. We found that some odors behave as immunostimulatory agents, whereas others might be considered as potential immunosuppressant odors. Interestingly, the immunomodulatory capacity was, in some cases, strain-specific. In particular, one of the fragrances, carvone, was found to be immunostimulatory in BALB/c mice and immunosuppressive in C57BL/6J mice, facilitating or impairing viral clearance, respectively, in a model of a viral infection with a recombinant adenovirus. Importantly, inhalation of the odor improved the memory capacity in BALB/c mice in a fear-conditioning test, while it impaired this same capacity in C57BL/6J mice. The improvement in memory capacity in BALB/c was associated with higher CD3^+^ T cell infiltration into the hippocampus and increased local expression of mRNA coding for IL-1β, TNF-α, and IL-6 cytokines. In contrast, the memory impairment in C57BL/6 was associated with a reduction in CD3 numbers and an increase in IFN-γ. These data suggest an association between the immunomodulatory capacity of smells and their impact on the cognitive functions of the animals. These results highlight the potential of studying odors as therapeutic agents for CNS-related diseases.

## Introduction

Humans and animals use the classic five senses to monitor their environment: sight, hearing, touch, smell, and taste. Their survival depends largely on the perception of these stimuli. Detecting situations through these senses transmits signals to the central nervous system (CNS) affecting the condition of the body and in turn promoting physiological changes in other biological systems.

The perception of external signals through the sense organs can significantly affect the immune system. The CNS and immune system are connected by bidirectional signaling pathways, so, changes in the CNS may affect immune function and *vice versa* (1–3) [and recently reviewed in Ref. (4–6)].

Smell has traditionally been considered a less important sense compared with sight or hearing, but recent studies have revealed valuable properties inherent to this sense, besides its importance for identifying food, family, predators, or dangerous situations. Human studies have shown that the perception of different smells influences the sympathetic and parasympathetic nervous systems and brain neurophysiological activity (7). Smells can also act on the neuroendocrine system, stimulating the production of neurotransmitters and neuromodulators, thus influencing psychological behavior and bodily functions (8). Olfactory system dysfunctions have been found in autoimmune diseases such as multiple sclerosis and other neurological disorders such as Alzheimer’s disease, Parkinson’s disease, schizophrenia, or depression ultimately related to alterations of the immune system, suggesting that, under certain circumstances, olfactory system abnormalities may be associated with the immune system [reviewed in Ref. (9)]. Experiments in animals subjected to olfactory bulbectomy show very significant effects on the immune system (10–12). It has also been reported that inhalation of different fragrances can regulate immunological reactions of the skin or may modulate the immune system (13, 14) and might have a therapeutic effect on diseases related to the immune system including CNS-related pathologies. All these findings indicate that the olfactory system has an all important connection to the immune system *via* the CNS, which should be explored.

As described by Buck and Axel (Nobel Prize, 2004), mammals have about 1,000 genes for odor receptors, 347 of which encode functional receptors (15). It is estimated that humans can smell more than 10,000 different odors. In contrast to other senses, such as sight, where we can define the colors based on the wavelength of light, in the case of smell, we lack a complete understanding of the organization of the spatial perception of odors (16). In a recent study, Castro et al. made a classification of odors into 10 minimum categories or clusters integrating a variety of descriptors or odors including: floral, woody, resinous, citrus, fruity non-citrus, chemical, minty or refreshing, sweet, burnt or smoky, sickening, or putrid (17). Using this classification to group odors into clusters, we performed an exploratory study of the immunomodulatory ability of a series of compounds representing each of the 10 categories. The aim of this project was to identify a volatile compound that, through stimulation of the olfactory system and the CNS, might have an effect on the activation of the immune system and eventually, on cognitive functions. We evaluated the impact of each particular odor on the immune response after immunization with ovalbumin (OVA) in combination with poly I:C adjuvant, a synthetic TLR3 agonist, which mimics a double-stranded RNA virus infection and favors dendritic cell maturation. We identified some odors with immunomodulatory properties that might be considered as therapeutic tools against different disorders. Interestingly, we also found an association between the immunomodulatory capacity of one of these odors with the number of CD3 T cells infiltrating the hippocampus, the hippocampal cytokine microenvironment, and its impact on the cognitive functions of the animals. These results might suggest a potential use of odors as therapeutic agents for CNS-related diseases.

## Materials and Methods

### Fragrance Compounds and Method for Delivery

All fragrance compounds were obtained from Aldrich Chemical Co., Milwaukee, WI, USA. Liquid and powdered compounds were dissolved in distilled water (1:200 v/v or 1:1,000 w/v, respectively). A closed system prototype was designed to allow the vaporization of fragrance compounds, which through a valve system controlled by a timer and a positive pressure equipment, allowed the independent inhalation of substances in different cages (Figure S1 in Supplementary Material). The central unit is the vaporization unit, which includes a ceramic ultrasonic vaporizer, immersed in a container with the aqueous solution to be vaporized. The vaporizer is located in an airtight container, which includes an air inlet to inject clean air and an outlet to recover the vaporized substance, which is then introduced into the cages housing the animals. A timer controls the operating time of the vaporizer. Inhalation of the fragrances was scheduled for different time periods (from 1 week to 1 month depending on the experiments), with 8 cycles of 15 min of inhalation per day. Air entry was aided by an air pump giving a constant flow of about 25 m^3^ of air/min. An outlet placed in the same cage allowed constant air exchange. When the vaporizer is inactive, the valve system allowed the entrance of clean air to the cages (25 m^3^ of air/min), thus ensuring continuous air renewal inside each cage.

### Mice and Experimental Design for *In Vivo* Experiments

Six-week-old BALB/c or C57BL/6 female mice (Envigo, Barcelona, Spain) were used to evaluate the impact of fragrance inhalation. This study was carried out in accordance with the institutional guidelines (CEEA, University of Navarra) and were approved by the institutional ethics committees (Ref R-109-14GN).

#### OVA Immunization Experiments

Naïve mice (*n* = 5) received an intravenous injection of OVA protein (1 nmol/mouse) plus poly I:C (50 μg/mouse). Seven days after immunization, splenocytes were obtained for immunological analysis. IFN-γ producing T cells were counted by ELISPOT using a kit from BD-Pharmingen (San Diego, CA, USA) following the manufacturer’s instructions. Briefly, plates (Multiscreen Filterplates. Millipore, Bedford, MA, USA) were coated with anti-IFN-γ AN18 antibody. After overnight incubation, the plates were washed with PBS and blocked for 2 h with RPMI containing 10% fetal bovine serum. Then, 10^6^ splenocytes/well were cultured in three replicates in the presence or absence of SIINFEKL peptide (1 µg/ml) [encoding the immunodominant H-2^b^ restricted CTL epitope (amino acids 257–264) from chicken OVA], peptide ISQAVHAAHAEINEAGR encoding the T helper epitope OVA (323–339), or OVA protein (10 µg/ml). In some experiments, anti-CD4 or anti-CD8 antibodies obtained from rat anti-mouse hybridomas GK 1.5 and H35.17.2, respectively, were added to the culture wells. One day later, plates were washed with PBS and incubated with biotinylated anti-IFN-γ R4-6A2 antibody and developed with freshly prepared 3-3′diaminobenzidine solution. The reaction was stopped with distilled water and spots were counted using an automated ELISPOT reader (CTL; Aalen, Germany). CTL activity was measured by an *in vivo* killing assay using target cells pulsed with SIINFEKL peptide as previously described (18). Briefly, syngeneic splenocytes were pulsed with OVA 257–264 peptide (10 µg/ml for 30 min at 37°C), washed extensively, and labeled with a high concentration (1.25 M) of CFSE. Another fraction of nonpulsed control splenocytes was labeled with a low concentration (0.125M) of CFSE. Both CFSE high- and CFSE low-labeled cells were mixed at a 1:1 ratio (5 × 10^6^ cells of each population) and then injected intravenously into the mice. Non-vaccinated mice were also immunized to normalize procedures across groups. The CFSE-labeled cells remaining in the spleen after 20 h was determined by flow cytometry as described previously. The ratio between IFN-γ-producing cells specific for OVA or SIINFEKL as well as the ratio of CTL activity specific for SIINFEKL peptide in splenocytes obtained from animals exposed to odor versus water vapor (air control) was calculated.

#### RAdLuc Inoculation Experiments

Naive mice (*n* = 10) were inoculated intravenously with 1 × 10^9^ pfu of a recombinant adenovirus expressing Luciferase (RAdLuc) (19). At day 4, 11, 18, and 25, animals were anesthetized and injected intraperitoneally with 150 mg/kg of d-luciferin. Five minutes later, they were placed in a darkroom (PHOTONIMAGER™, Biospace Lab) for light acquisition (exposure time 1 min). The quantification of the light emission was measured in photons/s/cm^2^/sr and quantified with the M3Vision™ Software.

For measurement of T cell immune responses induced by adenovirus inoculation, splenocytes were obtained at day 30 after RAdLuc injection and stimulation with heat-inactivated recombinant adenovirus. T cell proliferation was tested after 3 days of culture by measuring [methyl-3H]thymidine incorporation. Briefly, splenocytes were plated on 2 × 10^5^ cells/well and stimulated with the adenoviral particles (2 × 10^7^ heat-inactivated plaque forming units per milliliter of culture) for 3 days. On the second day of culture, 0.5 μCi of [methyl-3H] thymidine were added to each well and cells were incubated overnight. Cells were harvested (Filtermate 196 harvester; Packard Instrument, Meriden, CT, USA) and incorporated radioactivity was measured using a scintillation counter (Topcount; Packard Instrument) as a readout of T cell proliferation.

### Contextual Fear Conditioning

To evaluate the effect of carvone on cognitive function, a previously described fear-conditioning paradigm with minor modifications was used (20). Briefly, on day 1 (habituation), mice were placed in the training chamber for 3 min. Twenty-four hours later (training phase), mice were placed in the training chamber for 2 min. Subsequently, mice received a footshock (0.3 mA) lasting 2 and 30 s, later, mice were returned to their home cage. Long-term memory was evaluated during the test phase 24 h after training. In this case, mice were returned to the conditioning chamber and allowed to explore the context for 2 min, during which freezing behavior was recorded. Lack of movement except that required for breathing was defined as freezing. Freezing scores were expressed as percentages. The procedure was carried out in a StartFear system (Panlab S.L., Barcelona, Spain) that permits recording and analysis of the signal generated by the animal movement through a high-sensitivity Weight Transducer system. The analogical signal is transmitted to the FREEZING and STARTLE software modulated through the load cell unit for recording purposes and subsequent analysis in terms of activity/immobility.

### Isolation and Flow Cytometric Analysis of Immune Cells Infiltrating the Hippocampus

After 7 days of exposure to carvone or to air control, mice were sacrificed to analyze immune cell infiltration into the hippocampus. After transcardial perfusion, the hippocampus was isolated from both brain hemispheres and mechanically dissociated and digested with colagenase/DNAse. Myelin and cell debris were removed by percoll density gradient centrifugation. The resultant cells were then labeled with anti CD45-BV510, Ly6G-PE, CD3-APC, CD19-BV421, and Ly-6C-PCPC55 antibodies (BD-biosciences). Perfect-Count Microspheres (cytognos) were added for absolute cell counts. The composition of the brain infiltrate was determined by flow cytometric analysis as described previously (21). Briefly, the gating strategy was based on the analysis of brain-infiltrating leukocytes defined as CD45^high^. After gating the CD45^high^ population, polymorphonuclear neutrophils were identified by Ly-6G expression, while T lymphocytes were designated as CD45^high^ CD3^+^ cells. The remaining CD45^high^ cells were then distinguished by CD19 (B lymphocytes) and CD11b expression. The CD11b^+^ fraction was subclassified into Ly-6C^high^ “inflammatory monocytes” and a Ly-6C^low^ population that encompassed monocytes, dendritic cells (DC), and macrophages.

### mRNA Extraction and Measurement of Immune Gene Expression by Real-time PCR

Total RNA was extracted from the hippocampi and purified using the RNeasy^®^ Lipid Tissue Mini Kit (Qiagen). Purified RNA was reverse-transcribed and quantitative RT-PCR analysis was performed on an Applied Biosystems Prism 7900 System using Power SYBR^®^ Green PCR Master Mix (Applied Biosystems). The sequences of primers used in this study are given in Table S1 in Supplementary Material. β-actin was used as a reference housekeeping gene for normalization. Amplifications were carried out in three replicates and the relative expression of target genes was determined using the formula 2^ΔCt^ (ΔCt indicates the difference in the threshold cycle between β-actin and target genes).

### Statistical Analysis

Normality was assessed with the Shapiro–Wilk *W* test. Statistical analyses were performed using parametric (Student’s *t*-test and one-way ANOVA) and non-parametric (Mann–Whitney *U* and Kruskal–Wallis) tests. For all tests, a *P-*value <0.05 was considered statistically significant. Descriptive data for continuous variables are reported as mean ± SEM. GraphPad Prism was used for statistical analysis.

## Results

### Evaluation of the *In Vivo* Immunomodulatory Capacity of a Panel of Different Odors in C57BL/6 Mice

**Figure 1:**
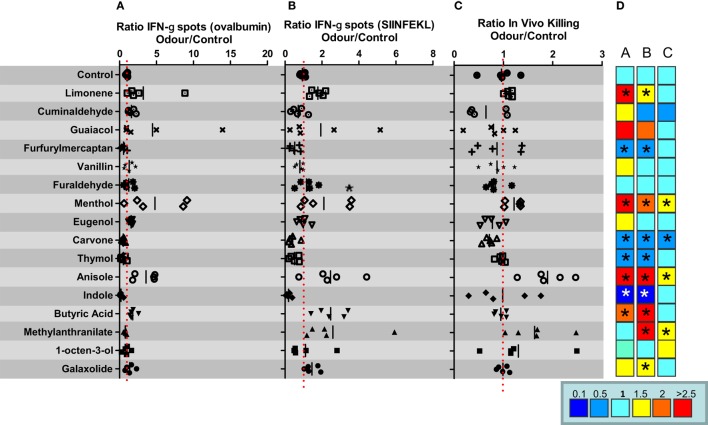
*In vivo* immunomodulatory capacity of a panel of different odors in C57BL/6 mice. C57BL/6 female mice (*n* = 5 mice per group) were immunized i.v. with ovalbumin (OVA) plus poly I:C. and 7 days after immunization IFN-gamma producing cells specific for SIINFEKL peptide **(A)** or OVA **(B)**, or the *in vivo* killing capacity specific for SIINFEKL peptide **(C)** was measured. **(D)** Heat map summarizing fold induction of the corresponding immune readout in mice exposed to the indicated compound versus mice exposed to water vapor. Data are representative of at least two independent experiments. *P < 0.05.

Following Castro et al., we selected a panel of 16 compounds representative of the 10 olfactory categories (17) to evaluate their impact on the immune response against injection of OVA in combination with poly I:C adjuvant. Thus, groups of five C57BL/6 female mice were immunized i.v. with 1 nmol of OVA mixed with poly I:C (50 μg/mice) in saline. After immunization, animals were housed in vaporization cages and exposed to the different odors for cycles of 15 min of odorization every 3 h during 7 days. Thus, animals were exposed to 8 cycles (15 min/cycle) of odorant vaporization per day. A control group was exposed to water vapor in the same conditions (air control). Seven days after immunization, animals were sacrificed and the immune response against OVA protein and the cytotoxic T cell epitope SIINFEKL was analyzed by ELISPOT to measure the number of IFN-γ-producing cells. The ratio between IFN-γ-producing cells specific for OVA or SIINFEKL in splenocytes obtained from animals exposed to odor versus water vapor was calculated. The capacity to induce SIINFEKL-specific CD8^+^ T cells was also measured by *in vivo* killing assays. The results of these experiments suggest that there are odors with immunomodulatory properties (Figure 1). Thus, odors such as limonene, menthol, anisole, methyl anthranilate, or butyric acid might behave as immunostimulators taking into account at least two of the three readouts used in this experiment (see heat map in Figure 1). These results appear to agree with previous results suggesting an immunostimulatory effect for limonene or menthol (22, 23). However, there are also reports assigning an immunosuppressive role to butyric acid in *in vitro* assays (24, 25), whereas in our hands, when it is inhaled, it could be considered immunostimulatory. On the other hand, furfuryl mercaptan, carvone, thymol, or indole might be considered potential immunosuppressant odors. Previous studies have also suggested an immunosuppressive effect for thymol (26, 27) or indole (28) in different settings, thus supporting the results shown in our experimental model of immunization.

The different immunomodulatory behavior of the molecules tested in C57BL/6 mice cannot be explained on the basis of the structural and pharmacophoric characteristics of the compounds (i.e., hydrogen-bond acceptor/donor, aromatic…). Indeed, clustering of the 16 compounds according to their similarity, determined by calculating FCFP_4 fingerprint using the Clustering Molecules component in Pipeline Pilot (Biovia, San Diego, CA) identified molecules as highly similar (FCFP_4 > 0.5) although they presented different biological profiles (e.g., limonene versus carvone and guaiacol versus vanillin; Table S2 and Figure S2 in Supplementary Material).

**Figure 2:**
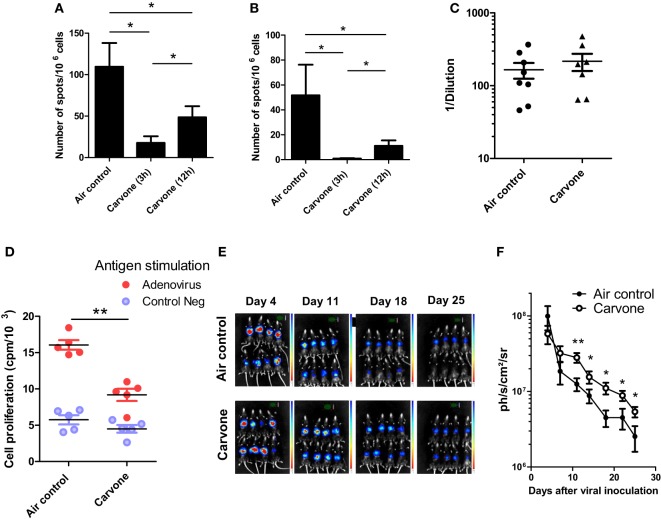
Effect of inhalation of carvone in the immune response against ovalbumin (OVA) **(A,B)** or RAd-Luc virus infection **(C–E)** in C57BL/6 mice. **(A,B)** Mice (*n* = 5) were immunized with OVA + poly I:C and exposed to the indicated schedules of carvone inhalation. Immune response to SIINFEKL **(A)** or OVA **(B)** after immunization with ovalbumin plus poly I:C and exposure to two different schedules of carvone inhalation (cycles of 15 min of exposure every 3 or 12 h) during 7 days (*n* = 5). **(C–E)** Mice were challenged i.v. with RAdLuc and exposed to carvone inhalation. **(D)** Cell proliferation of splenocytes in response to adenoviral particles in mice exposed to carvone or to air control (*n* = 5). **(E,F)** *In vivo* luciferase expression at different time points to evaluate the kinetics of viral clearance for each experimental group (*n* = 8). The mean value and the SEM are represented. Data are representative of two independent experiments. *P < 0.05; **P < 0.01.

We repeated the experiments with some of the odors of this selection and decided to study the effects of carvone in more detail, since it consistently behaved as an immunosuppressant in the OVA plus poly I:C vaccination experiment (data not shown). The effect of time of exposure to carvone was also tested in a simple assay using two different schedules: cycles of 15 min of odorization every 3 or 12 h during 7 days. Thus, C57BL/6 mice immunized with OVA + poly I:C were exposed to the two different odorization schedules. Seven days after immunization, we evaluated the immune response against OVA or SIINFEKL peptide by ELISPOT (Figures 2A,B, respectively; Figure S3 in Supplementary Material). We found that both schedules of carvone exposure induced immunosuppression against the antigen, although exposure to carvone every 3 h was significantly more immunosuppressive than exposure every 12 h. Thus, we decided to use this schedule for the rest of the experiments.

### Effect of Inhalation of Carvone in an Experimental Model of Viral Infection in C57BL/6 Mice

In order to study the immunosuppressive properties of carvone inhalation, we used an experimental model of viral infection. In this experiment, we infected female C57BL/6 mice with a recombinant adenovirus expressing luciferase (RAdLuc) through the tail vein to favor intrahepatic viral infection (29, 30). Following injection of the virus, mice were housed in isolated cages and exposed to carvone or water vapor (air control) as described in Section “Materials and Methods.” We measured the luciferase expression at different time points to evaluate the kinetics of viral clearance for each experimental group. In this model, luciferase expression decreases progressively from day 5 to day 25. At the end of the experiment, we measured T cell proliferation of splenocytes in response to viral particles, as a readout of antiviral T cell immune response. In agreement with the results shown in Figure 1, we found that mice exposed to carvone inhalation had a lower T cell proliferation capacity in response to stimulation with adenoviral particles as compared with that found in the control group (Figure 2C). Interestingly, carvone inhalation significantly delayed the decay in luciferase expression (Figures 2D,E,F; Figure S4 in Supplementary Material), a result consistent with the impairment of the activation of an antiviral immune response. This delay was statistically significant from day 10 to the end of the experiment. We also analyzed the effect of carvone inhalation during 3 weeks on the proportion of splenic CD3, CD4, CD8, CD19, CD11b, NK, NKT, and CD4^+^Foxp3^+^ cells but no significant changes were observed as compared with control group (Figure S5 in Supplementary Material).

### Effect of Inhalation of Carvone on Memory Capacity of C5/BL/6 Mice

**Figure 3:**
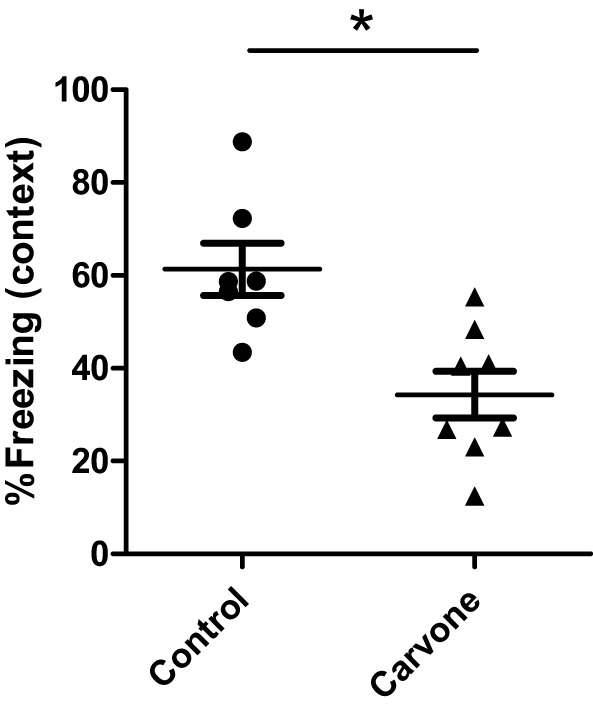
Percentage of freezing during the contextual FC test in C57BL/6 mice. Fear learning and memory was evaluated in mice immunized with ovalbumin (*n* = 6) and exposed to carvone or water vapor inhalation during 7 days using a classic fear-conditioning assay, as described in Section “Materials and Methods.” Data are representative of two independent experiments. *P < 0.05.

The olfactory system can have an impact on the CNS and on cognitive abilities. For this reason, we also studied the effect of exposure to carvone on memory capacity, using a fear-conditioning test or Freezing Test after 25 days of exposure to the odor. Using this method, we observed that mice exposed to carvone had a percentage of freezing values lower than those observed in mice exposed to water vapor (Air control), suggesting an impairment in their cognitive capacity (Figure 3).

### Effect of the Inhalation of Carvone on BALB/c Mice

**Figure 4:**
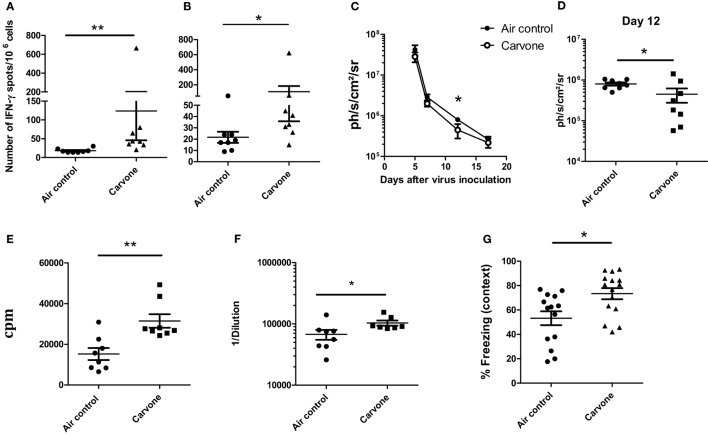
Effect of the inhalation of carvone in BALB/c mice. **(A,B)** Effect of carvone inhalation on the immune response [IFN-γ-producing cells specific for ovalbumin (OVA) **(A)** or for ISQ peptide **(B)**] after immunization with OVA. **(C–E)** (*n* = 8 mice per group). Effect of inhalation of carvone in viral clearance after RAd-LacZ virus infection **(C,D)** or in T cell proliferation in response to viral particles **(E)** (*n* = 8). **(F)** Anti-adenovirus antibody titers in the sera of the different groups of mice. **(G)** Percentage of freezing during the contextual FC test. Data from **(A,B)** are representative of three independent experiments. Data from **(C–G)** are representative of two independent experiments. *P < 0.05.

Experimental evidence suggests that mouse strains respond differently to odor stimulation (31). BALB/c mice had greater olfactory sensitivity than 129/S1 or C57BL/6 mice (32). There are also a large number of studies reporting differences in immune responses depending on the mouse strain. C57BL/6 mice are polarized to a Th1 immune response and BALB/c mice develop preferentially a Th2-type cytokine polarization (33–35). Since both C57BL/6 (H-2^b^) and BALB/c (H-2^d^) mice are commonly used for studies of immunoregulation in various disease models, we evaluated the effect of carvone on the immune response elicited after immunization with OVA plus poly I:C adjuvant in this second mouse strain. Surprisingly, in contrast to what occurred in C57BL/6 mice, carvone inhalation enhanced the immune response against the immunogen [measured by ELISPOT to determine the number of IFN-γ-producing cells specific for OVA protein or for the Th epitope ISQ (recognized by H-2^b^ and H-2^d^ T cells)] (Figures 4A,B, respectively). This result is consistent with a previous study showing that administration of carvone had immunostimulatory effects on BALB/c mice increasing antibody production (36).

When we studied the effect of inhalation of carvone or water vapor (air control) on the kinetics of RAdLacZ viral elimination in BALB/c mice, we found a slightly faster viral elimination in mice exposed to carvone (Figure 4C). This difference was statistically significant at day 12 after virus inoculation (Figure 4D). We also found that T cell immune response (T cell proliferation) and antibody production against adenoviral particles (Figures 4E,F, respectively) was higher in the group of mice exposed to carvone, all of which suggests that carvone is indeed functioning as an immunostimulatory odor in BALB/c as opposed to its immunosuppressive capacity found in C57BL/6.

Interestingly, when we performed the fear-conditioning test to evaluate the memory capacity of BALB/c mice, we found a significantly better behavior in mice exposed to carvone (Figure 4G).

### Effect of the Inhalation of Carvone on Leukocyte Infiltration in the Hippocampus of C57BL/6 and BALB/c Mice

**Figure 5:**
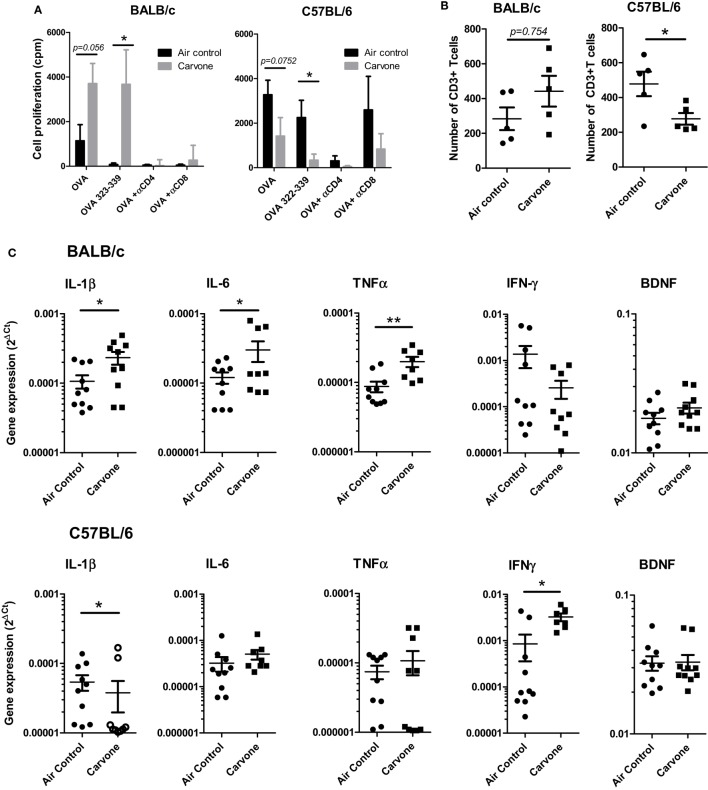
Effect of the inhalation of carvone in BALB/c and C57BL/6 mice. Mice were immunized with OVA + poly I:C and exposed to carvone or to air control inhalation for 7 days. **(A)** Effect of carvone inhalation on T cell proliferation of splenocytes in response to the indicated antigens and antibodies. **(B)** Effect on the number of CD3^+^ T cells infiltrating the hippocampus from BALB/c and C57BL/6 mice. **(C)** Quantitation of cytokine mRNA expression in the hippocampal tissue from BALB/c or C57BL/6 mice.

We evaluated whether exposure to carvone odor might affect the accumulation of immune cells in the hippocampus, leading to an effect on the local neuroinflammatory response and cognitive function. C57BL/6 or BALB/c mice were vaccinated with OVA plus poly I:C and exposed to carvone or to air control for 7 days. Using the splenocytes from these mice, we first confirmed the immunomodulatory activity of carvone in both strains of mice by measuring the proliferative capacity of splenocytes in response to OVA and the synthetic peptide from OVA 323–339, which is recognized as a T helper epitope by C57BL/6 and BALB/c mice. As expected, carvone was immunostimulatory in BALB/c and immunosuppressive in C57BL/6 (Figure 5A). Addition of anti-CD8 and especially, anti-CD4 antibodies abrogated T cell proliferation, suggesting that both T cell subpopulations are modulated by carvone inhalation.

In parallel, after transcardial perfusion to remove circulating leukocytes, hippocampi from the different groups of mice were isolated, dissected, and mechanically dissociated. After enzymatic dissection, infiltrating leukocytes were isolated and characterized as described in Section “Materials and Methods.” Interestingly, we found that in C57BL/6 mice, where carvone was acting as an immunosuppressant and where mice presented impaired cognitive function, the number of infiltrating CD3 T cells was significantly lower than in mice exposed to air control (*P* < 0.05). However, in BALB/c mice where carvone behaved as an immunostimulant and improved cognitive function, a tendency toward higher numbers of CD3 T cells infiltrating the hippocampus was observed (*P* = *0.754*) (Figure 5B). No significant changes were observed in the total numbers of polymorphonuclear leukocytes (CD45^high^ Ly6G^+)^, B cells (CD19^+^), monocytes, cDC, or macrophages (CD11b Ly6C^high^ or CD11b Ly6C^low^, respectively) (Figure S5 in Supplementary Material).

The processes of learning and memory can be affected by cytokines produced by immune cells (37, 38). We also isolated the mRNA from hippocampal tissue to analyze by RT-qPCR the hippocampal cytokine milieu. The cytokines IL-1β, IL-6, TNFα IFN-γ, IL-10, IL4, or BDNF, which have recently been shown to particpate in the induction of synaptic plasticity and changes in hippocampal-dependent learning and memory tasks [reviewed in Ref. (6)] were evaluated.

In our experimental conditions, we found that carvone induced a statistically significant increase in IL-1β, IL-6, and TNF-α in BALB/c mice. However, in C57BL/6 mice exposed to carvone, we found a reduction in IL-1β and an increase in IFN-γ (Figure 5C). No significant changes in BDNF were observed between either strains. IL10 and IL-4 were almost undetectable in both strains and thus were not further included in the study (data not shown). In Table S3 in Supplementary Material, we summarize all the main findings observed in both the C57BL/6 and BALB/c strains of mice.

## Discussion

Over the past years, it has become evident that the immune system plays a central role in learning, memory and neural plasticity, brain functioning, and behavioral processes [reviewed in Ref. (4)]. Under physiological conditions, immune mediators are induced by environmental/psychological stimuli and can participate on the regulation of the neural circuits remodeling, promoting learning, and memory consolidation [reviewed in Ref. (39)]. These beneficial effects of the immune system seem to be mediated by complex interactions among microglia and astrocytes (brain cells with immune functions), peripheral immune cells (mainly T cells and macrophages), neurons, and neural precursor cells [reviewed in Ref. (6)]. Neurotransmitters, hormones, inflammatory cytokines, as well as other mediators may also play a role in this remodeling. Infections, injuries, or severe or chronic stressful conditions can activate the immune system promoting the production of high levels of pro-inflammatory cytokines and other mediators that can influence behavior (5, 40, 41) and produce direct detrimental effects on memory, neural plasticity, and neurogenesis (42, 43) and consequently might have an important role in CNS diseases. Thus, inflammation has been associated with sickness behavior and infiltration of peripheral immune cells into the CNS with pathological results. However, several studies have pointed to neuroimmune interactions as being primarily beneficial, in that they promote homeostasis of the nervous system (4, 5, 41, 44–47). Accordingly, any intrinsic or environmental factors that might have an effect on the immune system may also have an impact on CNS behavior.

In this scenario of interacting networks, we have investigated the potential role that the olfactory system may have in the immune system and on the CNS. Indeed, several reports have documented immunomodulatory and neurological effects for several odorants. We have performed an exploratory study with 16 different compounds and found diverse effects on the activation of the immune system, with some compounds having an immunostimulatory activity whereas others behaved as immunosuppressants in C57BL/6 mice. One of the compounds identified as an immunosuppressant in C57BL/6 mice was carvone. Using this odor, we analyzed in mice the interaction between the three systems (olfactory, immune, and CNS) by measuring simple readouts such as the activation of an immune response against an antigen, induction of immune-mediated viral clearance, and the learning capacity of mice in an assay of contextual fear conditioning. Interestingly, carvone inhalation reduced the immunogenicity of OVA and delayed the capacity of C57BL/6 mice to clear viral infection. This immunosuppressive effect was also associated with a lower memory capacity (fear condition test). Curiously, carvone behaved as an immunostimulatory essence in BALB/c, enhancing the immunogenicity of OVA, favoring viral elimination and importantly, improving memory. When we analyzed the immune cells infiltrated into the hippocampus of the animals from both strains, we found that in C57BL/6 mice, where carvone was immunosuppressive and impaired cognitive function, the number of infiltrating CD3 T cells was significantly lower than in mice exposed to air control. However, in BALB/c, where carvone behaved as an immunostimulant and improved cognitive function, we found a tendency toward higher numbers of CD3 T cells infiltrating the hippocampus. It has been recently proposed that T lymphocytes may play a role in several neurodegenerative diseases such as Alzheimer’s disease and amyotrophic lateral sclerosis. Indeed, Alzheimer’s disease-susceptible mice progress to disease more rapidly in the absence of an adaptive immune system (48), suggesting that T cells may be protecting the diseased brain. Similarly, it has been reported that mice deficient in T lymphocytes exhibit cognitive impairment, and that passive transfer of mature T cells improves their cognitive function (49). These data lend support to our results in that when the odorant carvone induced a higher infiltration of CD3 T cell into the hippocampus, mice had better learning and memory behaviors.

The processes of learning and memory can be affected by cytokines produced by immune cells (37, 38). Thus, IL-1β, IL-6, TNF-α IFN-γ, IL-10, IL4, or BDNF have been described to play a role in the induction of synaptic plasticity and in changes in hippocampal-dependent learning and memory tasks [reviewed in Ref. (6)]. In our experimental conditions, we found that carvone induced a statistically significant increase in IL-1β, IL-6, and TNF-α in BALB/c mice. However, in C57BL/6 exposed to carvone, we found a reduction of IL-1β and an increase in IFN-γ.

Modulation of memory processes caused by these cytokines is a complex phenomenon having both facilitating and damaging effects depending on the specific proinflammatory cytokine or its levels in the brain (6, 50). There are recent reports showing that an excessive production of inflammatory Th1 cytokines such as IFN-γ have a deleterious effect on the brain (51). In other studies, IFNγ levels have been found to be increased in the brains and blood of individuals diagnosed with autism as well as in a mouse model of autism (52, 53). In contrast, it has been reported that proinflammatory cytokines such as IL-1β, IL-6, and TNF-α are involved in the processes of learning and memory (37, 38). Our data might be in line with those reports suggesting that lymphocytes and proinflammatory cytokines can modulate learning and memory through their effects on synaptic plasticity (54). However, how carvone odorant induces these immune changes in the hippocampal microenvironment and why these changes are different depending on the mouse strain remain to be elucidated.

Several reports have documented differences in sensitivity to odor stimulation depending on the mouse strain (31, 32) that may affect the cognitive functions and the response of the animals to an olfactory stimulation. These differences depending on the mouse strain background have been reported in other scenarios. C57BL/6 mice have been shown to respond with a TH1-type bias to pathogens, whereas other backgrounds of mice, such as DBA/2, BALB/c and A/J mice, tend toward a predominant TH2 response (33–35, 55). These differences are also observed for the M-1 and M-2 macrophage responses. Whereas TH1 cells are associated with intracellular pathogens clearance, TH2 cells are important against parasitic infections. TH1 or TH2 bias may affect outcomes after pathogen infections (56, 57). C57BL/6 mice, with TH1-biased responses, are in general more resistant to these organisms than other strains of mice. As a result of mutations and polymorphisms, inbred laboratory mouse strains are highly divergent in their immune response patterns. They present important differences in both the innate and the adaptive immune systems and may respond differently to the same stimulation (58).

Obviously, these differences on the immune status are expected to also occur in humans, where genetic variability, mutations, and polymorphisms are greater than in conventional animal models. These divergences can even be accentuated in humans if we take into account psychosocial and environmental factors that are not controlled as in animal experiments. This fact may limit our study and the desire to identify a particular odor for its potential use to stimulate a specific immunological or cognitive response. However, these experiments could shed some light on the understanding of the complex interaction between smell, the immune system, and the brain. Indeed, our results could be interpreted as being in agreement with recent reports suggesting that throughout lifetime, immune system supports cognitive function. It has been shown that disruption of the immune system leads to impairments in congnition and neurogenesis (44, 47) suggesting that an appropriate immunomostimulation by active immunization could protect against stressful episodes, providing a therapeutic/preventive vaccine against CNS-related diseases (59). It is tempting to speculate that a certain level of immunostimulation might favor brain plasticity enhancing adaptation, learning, and other functions of the CNS. In this scenario, aromatherapy, which has been widely but controversially employed for relief of pain, relaxation, anxiety reduction, reduction of postsurgical discomfort, or palliation of autoimmune diseases among others might also have a role to improve our mental fitness. However, further studies are required to assess this speculation more rigorously.

The mechanism of action by which odors can modulate the immune system and the CNS remains to be elucidated. Odorants could enter into the brain through the nasal epithelium bypassing the blood–brain barrier, thus gaining access to the CNS (60), and exerting direct pharmacological effects through olfactory receptors expressed in neurons (61). Alternatively, odors such as carvone could be transported to the CNS, through the cerebrospinal fluid (CSF) *via* the so-called glymphatic system, which could facilitate the spread of the odorant to multiple regions of the CNS (62–64). Another possible mechanism is that the odorant, after inhalation into the lungs, may enter the bloodstream and/or the lymphatic system and exert effects directly on the components of the immune system. There are also other variables that should be considered: odorant concentration, odorant mixtures, time of exposure, habituation, or other aspects such as spatial clustering of glomerular responses (16). Future studies should explore all these possibilities to help in the development of new therapeutic modalities targeting these systems.

## Ethics Statement

All experiments were performed following institutional guidelines and were approved by the institutional and local ethical committees (R-109-14GN).

## Author Contributions

This study was designed, directed and coordinated by JL with the collaboration of NC. MC-T, AG-O, KI, and MP-G provided conceptual and technical guidance for neurological aspects of the study and were implicated in the interpretation of data. PS and SH-S provided conceptual and technical guidance for immunological assays and were implicated in the interpretation of data. AL-C, NC, KI, and MG conducted most of the experiments of this study. The manuscript was written by JL; comments and edits were provided by all other authors (NC, TL, SH-S, KI, OR, MP-G, AL-C, MC-T, AG-O, and PS).

## Conflict of Interest Statement

The authors declare that the research was conducted in the absence of any commercial or financial relationships that could be construed as a potential conflict of interest.
