# Supplementary material for: Immunomodulatory Properties of Carvone Inhalation and Its Effects on Contextual Fear Memory in Mice
Source: Front Immunol. 2018 Jan 25;9:68. doi: 10.3389/fimmu.2018.00068 (PMC5788902; doi:10.3389/fimmu.2018.00068)
Supplement: Supplementary file 1 [file Presentation_1.PDF]

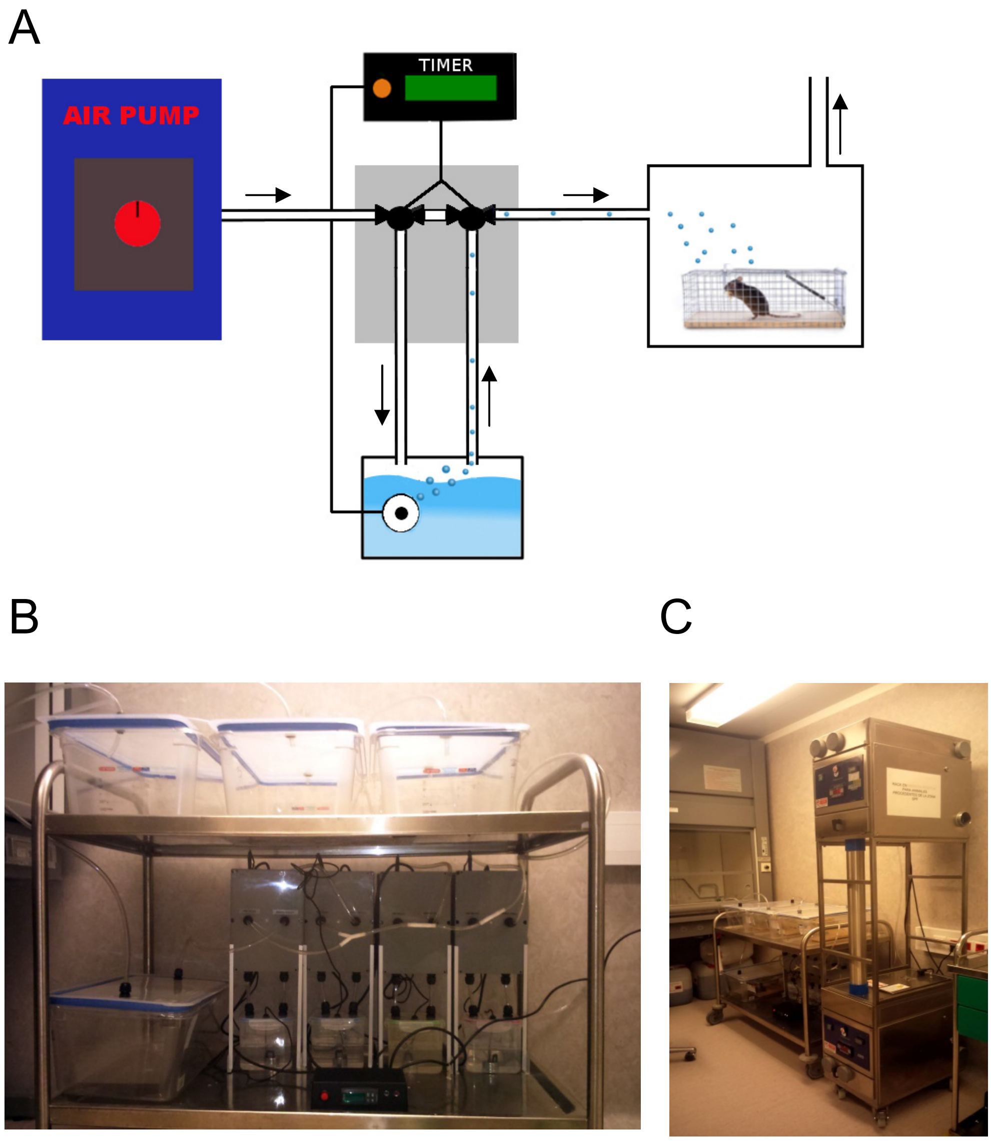

Supplementary Figure 1. Design of a closed system to allow the vaporization of fragrance compounds. (A) Schematic representation of the system. (B and C) Two photograps to illustrate the module with four independent vaporization units.

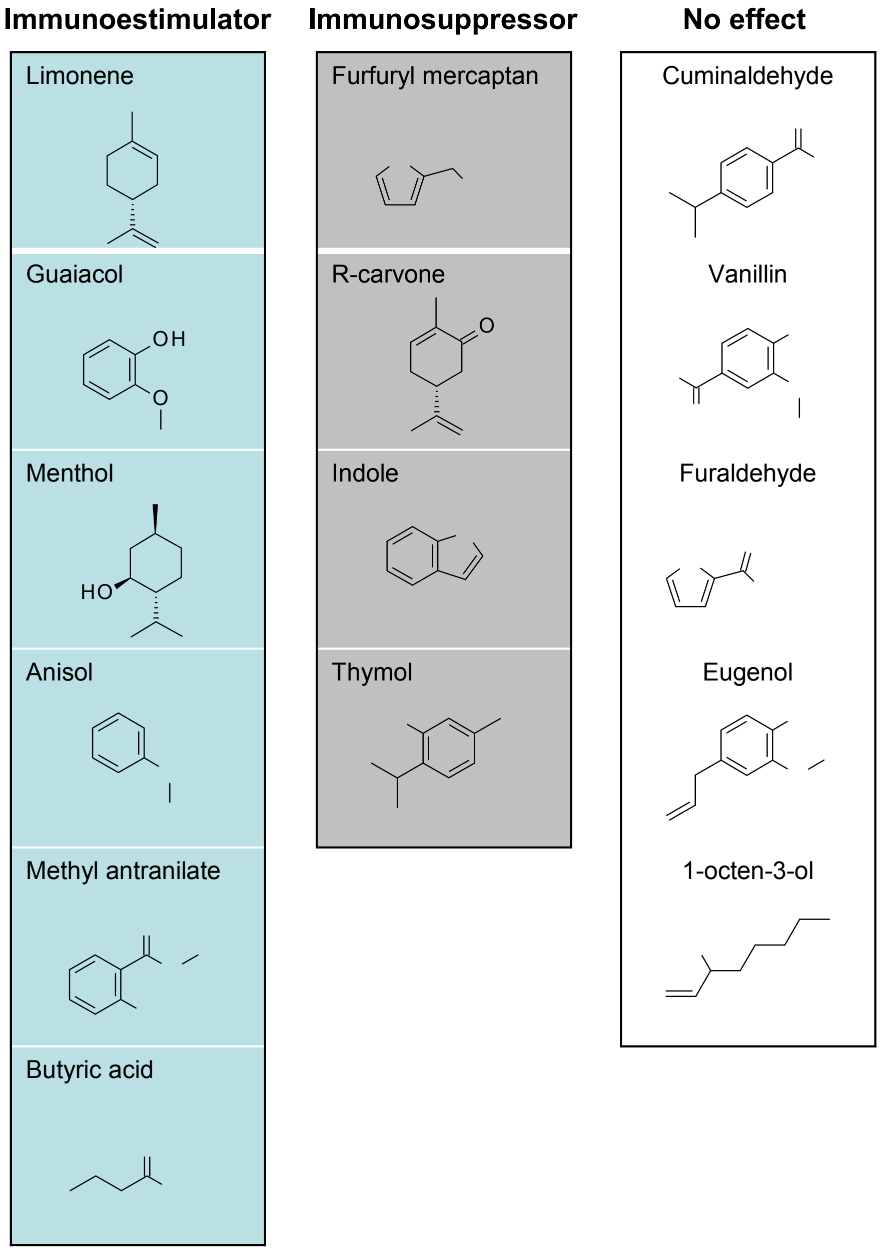

Supplementary Figure 2. Chemical structure of compounds tested. Molecules have been grouped according to their immunomodulatory activity.

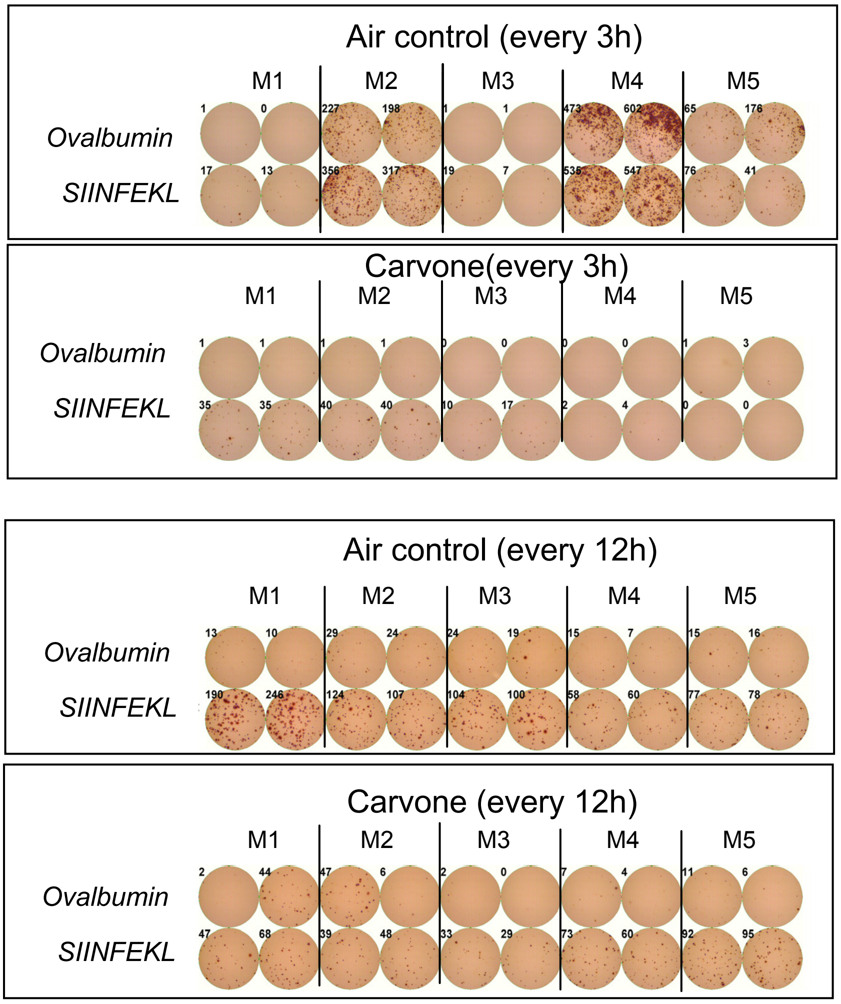

Supplementary Figure 3. Effect of carvone inhalation after immunization of mice with ovalbumin + poly I:C on the immune response against ovalbumin or SIINFEKL measured by ELISPOT (IFN-g producing cell spots). Pictures correspond to representative ELISPO of this assayM1-5 represents individual mice on each group. Mice were exposed to two different schedules: cycles of 15 min of odorization every 3h or every 12h during 7 days

A

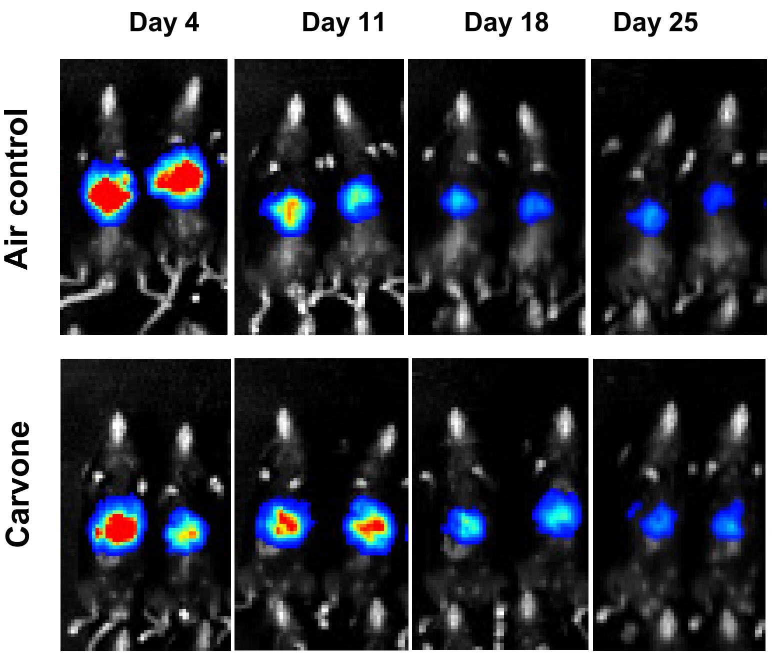

B

Days	Air control								Carvone							
4	2,40E+08	1,79E+08	1,37E+08	1,53E+08	8,88E+06	2,63E+07	4,96E+06	8,45E+07	1,12E+08	2,20E+07	1,72E+06	7,55E+07	5,62E+07	1,01E+08	9,39E+07	2,08E+06
7	5,03E+07	6,63E+05	2,67E+07	2,17E+07	2,99E+07	6,30E+05	1,63E+07	1,75E+06	5,92E+07	7,12E+07	2,82E+07	2,03E+07	1,39E+07	2,78E+07	2,71E+07	8,78E+06
11	2,64E+07	1,46E+07	1,40E+07	1,13E+07	1,04E+07	2,21E+06	7,77E+06	1,35E+07	4,10E+07	4,49E+07	3,36E+07	1,84E+07	1,58E+07	3,38E+07	2,28E+07	1,46E+07
14	1,80E+07	8,88E+06	1,15E+07	6,13E+06	8,06E+06	2,94E+06	9,20E+05	5,96E+06	2,60E+07	1,95E+07	2,67E+07	7,85E+06	1,83E+07	7,89E+06	8,26E+06	9,78E+06
18	9,02E+06	6,29E+06	6,20E+06	7,47E+06	2,37E+06	5,49E+05	1,18E+06	2,86E+06	1,50E+07	1,75E+07	1,75E+07	6,24E+06	5,43E+06	1,46E+07	5,73E+06	6,53E+06
25	6,95E+06	3,17E+06	5,13E+06	3,94E+06	2,21E+05	6,08E+05	8,97E+04	1,39E+05	7,75E+06	7,43E+05	6,92E+06	4,08E+06	1,40E+06	7,50E+06	4,62E+06	5,44E+06

Supplementary Figure 4. Effect of inhalation of carvone in viral clearance kinetics. Female C57BL/6 mice were inoculated intravenously with a recombinant adenovirus expressing luciferase (RAdLuc) to favour intrahepatic viral infection. Luciferase expression was measured at different time points to evaluate the kinetic of viral clearance (A) Represetative examples of luciferase signal obteined in two mice exposed to air control or to carvone inhalation. (B) Raw data (Photons/sec/cm^2) obtained for each animal at different time points.

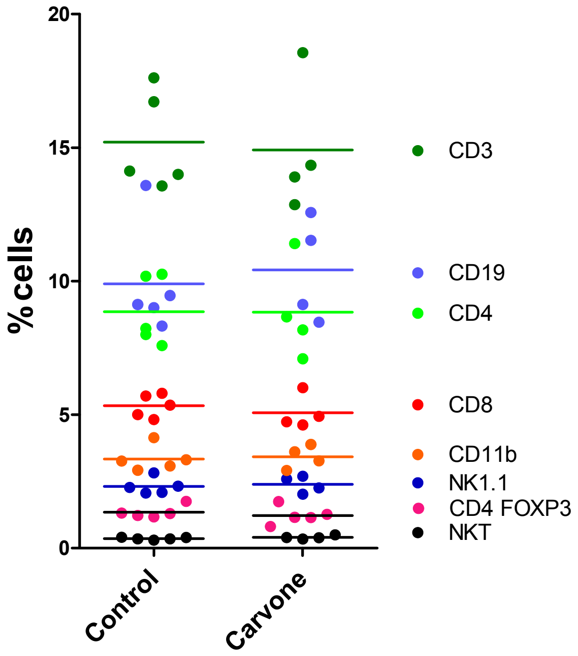

Supplementary Figure 5. Effect of carvone inhalation during three weeks on the percentage numbers of CD3, CD4, CD8, CD19, CD11b, NK, NKT and CD4+Foxp3+ cells in the spleen.

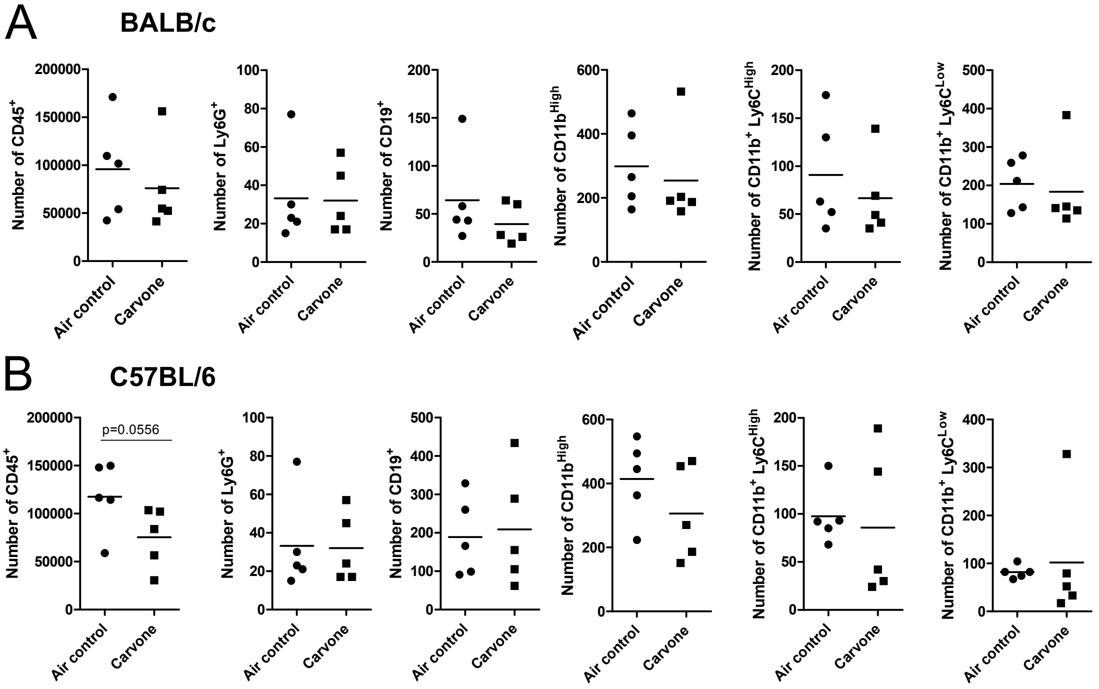

Supplementary Figure 6. Effect of carvone inhalation on the numbers of different leukocyte subpopulations infiltrating the hippocampus of BALB/C mice (A) or C57BL/6 mice (B) immunized with ovalbumin + Poly I:C and exposed to carvone or to air control.

Supplementary Table 1. Sequence of primers used to measure cytokine mRNA expression.

Gene	Sense primer (5′ –3′)	Antisense primer (5′ –3′)
IL-1b	GCCACCTTTTGACAGTGATG	TAATGGGAACGTCACACACC
IFN- γ	TCAAGTGGCATAGATGTGGAA	TGGCTCTGCAGGATTTTCATG
IL-10	GGACAACATACTGCTAACCG	AATCACTCTTCACCTGCTCC
IL6	ACAAAGCCAGAGTCCTTCAG	TGGATGGTCTTGGTCCTTAG
IL4	GCTATTGATGGGTCTCAACC	TCTGTGGTGTTCTTCGTTGC
TNF-α	CTTCCAGAACTCCAGGCGGT	GGTTTGCTCGACGTGGGC
BDNF	CTCCTCTACTCTTTCTGCTG	CCACTCGCTAATACTGTCAC
β-Actin	CGCGTCCACCCGCGAG	CCTGGTGCCTAGGGCG

Supplementary Table 2: Chemical similarities between compounds. Compouds have been classified as immunostimulatory, inhibitory or without effect according to the results obtained in C57BL76 mice and summarized in Figure 1.

	Immunostimulatory						Inhibitory				No effect				
CHEMISTRY	Lim	Gua	Men	Ani	MeA	BuA	FuM	Car	Ind	Thy	CuA	Van	FuA	Eug	Oct
Lim	1,00	0,03	0,23	0,04	0,03	0,14	0,03	0,55	0,00	0,07	0,08	0,03	0,09	0,23	0,23
Gua	0,03	1,00	0,04	0,59	0,21	0,13	0,14	0,06	0,11	0,23	0,15	0,61	0,14	0,56	0,04
Men	0,23	0,04	1,00	0,04	0,07	0,14	0,04	0,15	0,00	0,07	0,08	0,03	0,04	0,03	0,30
Ani	0,04	0,59	0,04	1,00	0,25	0,10	0,17	0,08	0,13	0,13	0,19	0,33	0,17	0,31	0,04
MeA	0,03	0,21	0,07	0,25	1,00	0,11	0,13	0,09	0,10	0,17	0,27	0,25	0,17	0,20	0,07
BuA	0,14	0,13	0,14	0,10	0,11	1,00	0,08	0,22	0,00	0,13	0,19	0,14	0,13	0,10	0,20
FuM	0,03	0,14	0,04	0,17	0,13	0,08	1,00	0,06	0,07	0,10	0,15	0,12	0,60	0,11	0,04
Car	0,55	0,06	0,15	0,08	0,09	0,22	0,06	1,00	0,00	0,06	0,15	0,09	0,10	0,11	0,20
Ind	0,00	0,11	0,00	0,13	0,10	0,00	0,07	0,00	1,00	0,07	0,07	0,06	0,07	0,06	0,00
Thy	0,07	0,23	0,07	0,13	0,17	0,13	0,10	0,06	0,07	1,00	0,36	0,28	0,10	0,26	0,07
CuA	0,08	0,15	0,08	0,19	0,27	0,19	0,15	0,15	0,07	0,36	1,00	0,40	0,25	0,19	0,08
Van	0,03	0,61	0,03	0,33	0,25	0,14	0,12	0,09	0,06	0,28	0,40	1,00	0,19	0,63	0,03
FuA	0,09	0,14	0,04	0,17	0,17	0,13	0,60	0,10	0,07	0,10	0,25	0,19	1,00	0,11	0,04
Eug	0,23	0,56	0,03	0,31	0,20	0,10	0,11	0,11	0,06	0,26	0,19	0,63	0,11	1,00	0,09
Oct	0,23	0,04	0,30	0,04	0,07	0,20	0,04	0,20	0,00	0,07	0,08	0,03	0,04	0,09	1,00

Abreviations: **Lim**: Limonene; **Gua:**Guaiacol; **Men**:Menthol; **Ani**: Anisole; **MeA**: Methyl antranilate; **BuA**: Butyric Acid; **FuM**: Furfuryl mercaptan; **Car**: Carvone; **Ind**: Indole; **Thy**: Thymol; **CuA**: Cuminaldehyde; **Van**:Vanillin; **FuA**: Furaldehyde; **Eug**: Eugenol; **Oct:** 1-octen-3-ol

Supplementary Table 3. Summary of main findings observed in both the C57BL/6 and BALB/c strain of mice.

**CARVONE versus AIR CONTROL**			**C57BL/6**	**BALB/c**
SPLEEN	ELISPOT (IFN-γ)		↓	↑
	ADENOVIRAL ELIMINATION		↓	↑
	PROLIFERATION		↓	↑
HIPPOCAMPUS	CD3+ CELL INFILTRATION		↓	↑
	RT-PCR	IFN-γ	↑	↓
		TNF-α	-	↑
		IL-1β	↓	↑
		IL-6	-	↑
FEAR CONDITIONING			↓	↑
